# The Expansion of Cholangioscopy: Established and Investigational Uses of SpyGlass in Biliary and Pancreatic Disorders

**DOI:** 10.3390/diagnostics10030132

**Published:** 2020-02-29

**Authors:** Michael Yodice, Joseph Choma, Micheal Tadros

**Affiliations:** 1 Albany Medical College, Albany, NY 12208, USA; yodicem@amc.edu; 2 Albany Gastroenterology Consultants, Albany, NY 12206, USA; jchoma@albanygi.com

**Keywords:** cholangioscopy, pancreatoscopy, difficult bile duct stones, indeterminate strictures, primary sclerosing cholangitis, SpyGlass

## Abstract

Direct visualization of bile and pancreatic duct pathology is proving to be beneficial in patients where previous techniques have failed. Recent advancements in technology and the development of the SpyGlass system have led to an increased use of cholangioscopy. It is already known that SpyGlass is beneficial in patients with difficult bile duct stones and indeterminate biliary lesions through the use of targeted lithotripsy and visually guided biopsy. Cholangioscopy allows the visualization of hidden stone and guide wire placement across difficult strictures and selective cannulation of the intrahepatic and cystic ducts. It is also demonstrating its utility in investigational applications such as post-liver transplant and primary sclerosing cholangitis stricture treatment, evaluation of hemobilia, and guided radiofrequency ablation of ductal tumors. In addition to having clinical utility, cholangioscopy may also be cost-effective by limiting the number of repeat procedures. Cholangioscopy overall has similar complication rates compared to other standard endoscopic retrograde cholangioscopy (ERCP) techniques, but there may be higher rates of cholangitis. This could be mitigated with prophylactic antibiotic use, and overall, cholangioscopy has similar complication rates compared to other techniques.

## 1. Introduction

Endoscopic retrograde cholangioscopy (ERCP) is currently the tool of choice for diagnosis and intervention in pancreatobiliary disease. While this technique is successful for many different scenarios, it remains limited by the fact that the endoscopist is only able to visualize structures indirectly via fluoroscopy. This indirect visualization can be especially limiting in patients with larger biliary stones and indeterminate biliary strictures [1]. In addition, other ERCP techniques such as brush cytology and biopsy are limited by a low sensitivity for the detection of malignant lesions [2]. Direct peroral visualization of the bile duct has been available since the 1970s, but only recently has the technology improved enough to show diagnostic and therapeutic benefit when previous techniques have failed.

There are currently three systems available for cholangioscopy including single operator, dual operator, and direct cholangioscopy. Advances have been made in all of these systems, with each one having certain advantages, such as ease of use, better image quality, or other diagnostic options like narrow band imaging [3]. This review will focus specifically on the recent advances in single operator cholangioscopy, which has allowed for easier use and wider utilization.

The initial peroral cholangioscope utilized a “mother-daughter” system in which a fiberoptic scope was passed through a duodenoscope before entering the bile duct [4]. The instrument, however, was bulky, required two trained endoscopists, and had poor image resolution (Table 1). Technological improvements in cholangioscopy continued over the years with the development of the SpyGlass system (Boston Scientific Corporation, Natick, MA, USA) in 2007. The first-generation SpyGlass was a reusable single operator fiberoptic scope with a “mother-baby” system including a channel for accessory instruments and irrigation capabilities. It had a 3.3 cm outer working diameter and a length of 230 cm that allowed for use with a standard duodenoscope. It included a 4-way deflectable tip allowing for greater maneuverability and easier use [5]. It also included dedicated accessory and irrigation channels, with the accessory port allowing for use of instruments like biopsy forceps and electrohydraulic or laser lithotripsy probes. In 2015, the second-generation SpyGlass DS was developed. This improved upon the first-generation by providing a digital image with 4x greater resolution and a wider field-of-view (110° vs. 70°). In addition, it also included a redesigned accessory channel for easier use and was designed for quick and simple setup in each case [6]. A recent study comparing the outcomes of fiberoptic and digital cholangioscopy found a significant increase in utilization rates of digital cholangioscopy [7], likely indicating that the increases in technology allowed for wider use of SpyGlass.

Single operator cholangioscopy (SOC) offers many benefits over standard ERCP due to the direct visualization of the pancreatic and bile ducts (Table 2). It has already been established that SOC is clinically beneficial for patients with difficult bile duct stones and indeterminate biliary lesions [8], and with increasing technology and ease of use, SOC is being used in more investigational applications as well (Table 3). This review will discuss the current established applications of SOC as well as some newer diagnostic and therapeutic uses.

## 2. Biliary Stones

**Figure 1:**
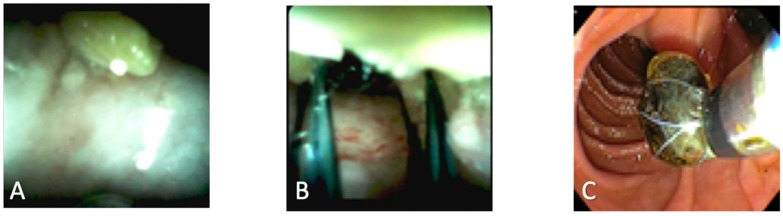
(**A**) Intrahepatic bile duct stone; (**B**) Positioning of the SpyGlass basket accessory around bile duct stone; (**C**) Removal of bile duct stone using SpyGlass basket accessory.

Recent studies have shown the benefit of using SOC in patients with both difficult and missed bile duct stones (Figure 1). Stones can be classified as difficult when they are larger in size (>15 mm), impacted, located in a difficult locations such as near strictures, or if there are multiple stones present [9]. Various locations in the bile duct network have also been described where stones may be hidden or missed from detection using magnetic resonance cholangiopancreatography (MRCP) and ERCP [10]. Stones impacted in the cystic duct or intrahepatic ducts, or adherent to the wall, can be difficult to visualize on standard cholangiogram and may go undetected. In these circumstances, direct visualization of the stone through SOC can allow for greater success in treatment and removal of the stone by guiding wire placement [10]. Patients with recurrent cholangitis, significant dilation of the bile ducts, and with unusual biliary stone presentation may also benefit from direct bile duct visualization with spy glass [10].

In addition to the benefits of directly visualizing a stone, SOC also allows for other treatment options such as laser or electrohydraulic lithotripsy. Previous methods available for the fragmentation of difficult stones included extracorporeal shockwave lithotripsy or ERCP guided laser lithotripsy [11]. These techniques were again limited by the lack of direct visualization of the stone and the reliance of indirect fluoroscopy to guide laser placement. With cholangioscopy, the difficult stones are fully visualized allowing for accurate laser targeting of the stone for successful fragmentation.

In 2016, a study looked at the usefulness of the first-generation SpyGlass for biliary disease in a single center cohort of 39 patients. The researchers found that cholangioscopy allowed for successful clearance in 82.1% of patients with difficult biliary stones. Most of these patients had stones in areas where the SpyGlass system provided helpful visualization such as the extrahepatic and proximal intrahepatic biliary tree. The researchers found that stones deeper in the intrahepatic ducts became more difficult to properly assess and led to failed procedures [12].

Another recent retrospective study also assessed the use of SpyGlass DS on patients found to have difficult bile duct stones. The outcomes were studied in 407 patients from multiple centers who underwent SOC with electrohydraulic or laser lithotripsy. Technical success was defined as complete clearance of stones and was found in 97.3% of patients. In addition, 77.4% of patients required only one session of SOC to achieve full ductal clearance. Further analysis showed difficult anatomy was a significant predictor of outcome failure [13]. Overall, SOC was found to be a safe and effective alternative to more invasive treatment approaches.

In 2019, a similar study was conducted with the primary endpoint of stone clearance in a single session of SOC. The study included 156 patients with 80% having failed stone clearance on previous ERCP attempts. Overall, the group found that cholangioscopy allowed for direct visualization of stones in 100% of patients, and that stone clearance was successful in a single SOC procedure for 80% of cases. The stones were cleared using either electrohydraulic or laser lithotripsy, with no significant difference in outcomes between the two (74% vs. 82%) The use of SpyGlass also had a significant impact on patient management, leading to a change in diagnosis or therapy in 91% of patients, including 83 patients who no longer needed surgical stone removal [14]. 

## 3. Indeterminate Biliary Strictures

**Figure 2:**
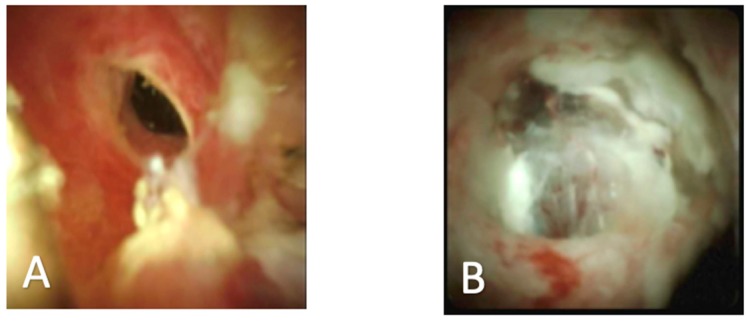
(**A**) Benign inflammatory bile duct stricture; (**B**) Stricture formation with malignant transformation.

In addition to utility in bile duct stones, recent studies have also demonstrated the benefits of cholangioscopy in the evaluation of indeterminate biliary lesions. Correct diagnosis of benign versus malignant biliary stricture is important for clinical decision making and patient management. It is known that the usage of ERCP with brush cytology and biopsy have a low sensitivity for detecting malignant disease. A recent review reported the sensitivity of cytologic brushing at 45% and biopsy at 48.1%. When these techniques are combined, the sensitivity improves to just 59.4% [2]. Recent studies have also shown that up to 24% of patients have surgery to resect a biliary stricture that is later found to be benign [15]. Direct visual inspection of bile duct strictures may help the determination of benign versus malignant stricture and may also allow for targeted and accurate tissue biopsy (Figure 2).

It is known that there are specific visual findings unique to different types of bile duct tumors. Malignant tumors have been described as having features of neovascularization, papillary projections, and tortuous vessels. Tumors that are benign are found to include tissue with ulceration and band like strictures with smooth mucosa [16]. Using cholangioscopy for direct visualization of the bile duct tissue along with targeted biopsies of the lesions can help improve the sensitivity of determining the malignancy of a growth. A recent systematic review assessed if SOC was useful for the diagnosis of indeterminate biliary lesions. Researchers reviewed 8 studies including 335 patients and found using cholangioscopy for visually guided biopsy was 69% sensitive and 98% specific for determining malignancy. In addition, they found that visual inspection of the bile duct was 90% sensitive and 87% specific for malignancy [17]. Another recent systematic review assessed 10 studies with 456 patients and found visually guided biopsy leads to a moderate improvement in sensitivity at 60%, and found specificity to be 98% [18]. These results show the utility of visual inspection for indeterminate biliary strictures, but the lack of a standardized visual classification system is limiting, and visual findings still should be confirmed by biopsy.

In addition to helping in the evaluation of patients with biliary strictures, cholangioscopy can also help guide further clinical management. A recent prospective study assessed the impact of SOC on the management of patients with indeterminate biliary lesions. The group found similar sensitivities and specificities with using SpyGlass in determining stricture malignancy, and also found SOC significantly altered patient management in patients with a challenging diagnosis. Visual inspection and biopsy of tissue that appeared to show inflammation helped to guide clinical decisions toward more conservative treatment in patients who would otherwise undergo surgery [19]. Again, this study was limited by the lack of standardized visual classification system, but visual inspection followed by target biopsy can allow for better clinical management in patients with biliary strictures.

Cholangioscopy may also be useful in patients with a biliary stricture already diagnosed as cholangiocarcinoma. A recent pilot study evaluated the use of SOC as a pre-op tool to assess the extent of tumor involvement. Patients already diagnosed with extrahepatic cholangiocarcinoma underwent SOC to obtain further biopsies to map the area of tumor involvement before surgical resection. The use of SpyGlass was found to be beneficial in these patients with 88% of biopsies showing malignant tissue [20]. Visually guided biopsy allows for more targeted tissue acquisition and the increased resolution of SpyGlass DS allows the user to specifically target tissue they believe is malignant. 

One recent issue that has developed with the increasing use of cholangioscopy is the difficulty of processing the small biopsy samples. A recent study evaluated the use of cell block cytology, which is used for processing smaller specimens, versus standard histopathology of tissue obtained using SpyBite biopsy. Researchers found that there was no significant difference between the two techniques, and observed that cell block cytology tended to have less artifact secondary to crushing [21]. Another recent study evaluated the number of biopsies necessary to find a definitive diagnosis using SpyBite. Patients were also randomized to have tissue samples processed at onsite vs. offsite labs. Researchers found no significant difference in diagnostic accuracy based on where the samples were processed (90% onsite vs. 87.5% offsite), and also found that three biopsies led to 90% diagnostic accuracy [22].

## 4. Cholangioscopy in Primary Sclerosing Cholangitis

The direct visualization offered by cholangioscopy may be beneficial in patients with primary sclerosing cholangitis (PSC). Patients with PSC are at a higher risk of developing complications such as benign strictures or cholangiocarcinoma [23]. Regular surveillance is important in these patients but there is a lack of non-invasive testing with a high sensitivity for detecting malignant lesions [24]. As previously discussed, visualization with cholangioscopy and guided biopsy may help but is limited by the lack of standardized visual classification system.

A recent study attempted to address this limitation by developing a novel classification system for bile duct strictures specifically in patients with PSC. The goal of the study was to better classify inflammatory strictures that can be seen in PSC and create a standardized system that could be used to monitor disease progression and guide therapy. The study included 30 patients with PSC and a bile duct stricture and classified these patients into three phenotypes based on visual features: inflammatory type, fibro-stenotic type, or nodular mass-forming type. The phenotypes could then be used to determine a patient’s risk for malignancy and help guide further management [25]. This study again shows the advantage that direct visualization can have compared to traditional ERCP techniques on patients with biliary strictures. The authors of this study are currently working on correlating the visual findings on cholangioscopy with histopathology from biopsy. They also acknowledge that larger scale studies are needed to further develop a management algorithm that can be used in patients with PSC [25].

## 5. SpyGlass for Pancreatic Pathologies

**Figure 3:**
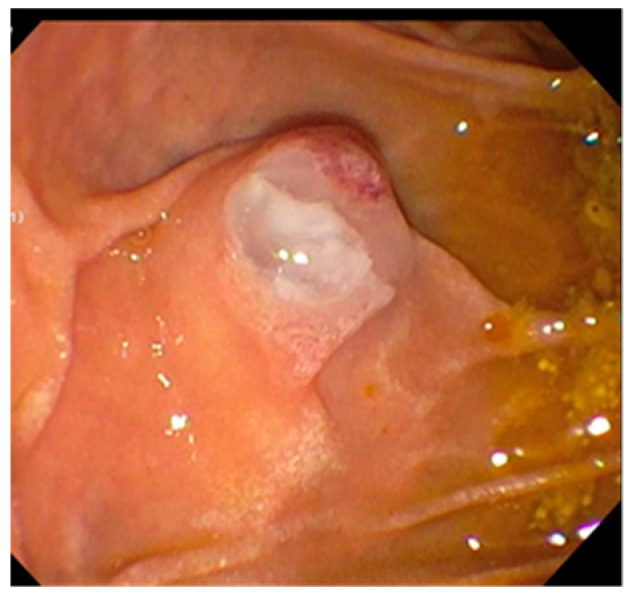
Intraductal papillary neoplasm of the pancreatic duct.

Like cholangioscopy, SpyGlass can be used in pancreatoscopy for similar diagnostic and therapeutic applications. SpyGlass may be beneficial specifically in the visual identification of intraductal papillary neoplasm, assessment of pancreatic strictures, and visualization and removal of pancreatic duct stones (Figure 3). Previous studies have established a visual classification system for the evaluation of benign versus malignant intraductal pancreatic tumors. The presence of these specific visual features was found to be 68% sensitive for malignancy [26]. A recent 13-year study evaluated the use of pancreatoscopy in the assessment of pancreatic duct tumors. The study included 79 patients who underwent pancreatoscopy for evaluation of a pancreatic duct lesion. Overall, visual impression of the lesion was found to be 87% sensitive for the detection of neoplastic growth. This improved to 91% when combined with visually guided tissue biopsy. In 97% of patients, technical success was achieved with successful advancing of the scope to the lesion, full visualization of the lesion, and proper diagnostic maneuvering. Adverse events were observed in 12% of cases, with most patients experiencing post-procedure abdominal pain and only 4% of patients developing post-procedure pancreatitis [27]. The authors concluded that pancreatoscopy can be highly successful and should be considered in patients with difficult pancreatic pathologies. 

**Figure 4:**
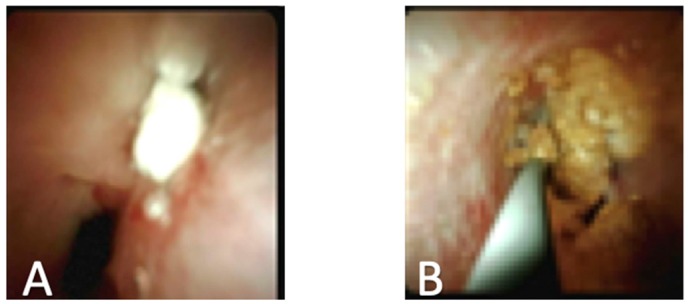
(**A**) Pancreatic duct stone; (**B**) Passing of wire by pancreatic duct stone.

Pancreatoscopy may also have benefit in patients with pancreatic duct stones (Figure 4). While there is more data available on outcomes of cholangioscopy for bile duct stones, similar techniques such as the use of electrohydraulic and laser lithotripsy may be useful for pancreatic stones. A recent study looked at laser lithotripsy in patients with chronic pancreatitis due to pancreatic duct stones. The authors found that 79% of patients had full stone clearance after visually guided laser lithotripsy. In addition, 89% of patients had clinical success defined as improvements in pain, decreased narcotics use, and decreased hospitalizations. This clinical success was found even though some patients required repeat pancreatoscopy or ERCP to remove further stones, indicating the benefits of pancreatoscopy compared to previous techniques [28]. 

## 6. Expanding Options for Treatment of Post-Transplant Stricture Treatment

With technological improvements and increased ease of use, SOC is being used in more investigational applications as it becomes more widely available. Cholangioscopy is being used in more situations where direct visualization of pancreatobiliary pathology could help with diagnosis or treatment. One scenario involves the use of SOC to manage patients after liver transplantation. The development of benign strictures is a complication that must be monitored after a liver transplant. When stricture does occur, there is no standard management protocol, but it is typically treated with balloon dilation or stenting. One study assessed the use of cholangioscopy in patients with post-transplant anastomotic strictures. Technical success was achieved in all patients and direct visualization allowed for easier balloon dilatation. In addition, cholangioscopy allowed for targeted steroid injections that aided in treatment of the strictures [29]. While this study was limited by a small sample size, it demonstrates the therapeutic potentials that direct visualization through cholangioscopy allows. 

**Figure 5:**
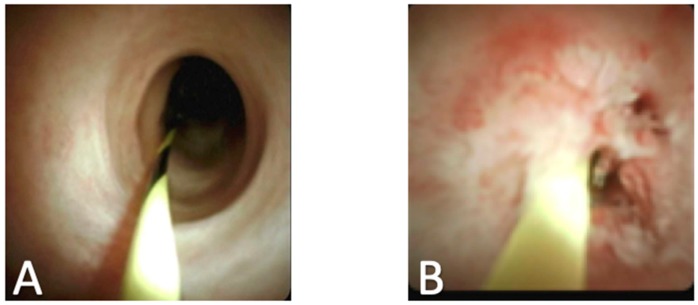
(**A**) Bile duct guidewire; (**B**) Passage of guidewire through stenosed bile duct.

Another potential treatment for patients with post-transplant biliary stricture development is the placement of ductal stents. In order to properly place the stent, a guidewire must first be passed through the stricture (Figure 5). This can be difficult in areas that are highly fibrotic and stenosed and may not be possible with conventional ERCP. A recent case series reported on using SOC in post-transplant patients where ERCP stenting had previously failed. The authors explained that direct visualization of a tiny opening in the stricture was the only way to successfully pass the guidewire. Once the guidewire had passed, proper stent placement was achieved and the patients were able to avoid surgical treatment of their strictures [30]. Visually assisted guidewire placement may also be beneficial in other biliary strictures besides post-transplant complications. In 2019, a retrospective study assessed the use of cholangioscopy in 30 patients with complex strictures who previously failed guidewire placement using conventional ERCP. It was found that cholangioscopy was 70% successful in guidewire passage and this increased to 88.2% if the stricture was benign in nature [31]. This study also shows the utility of cholangioscopy as an alternative to more invasive surgical treatment.

## 7. Management of Cholangiocarcinoma

Recent case reports have also described the benefits of SOC in the treatment of cholangiocarcinoma. In addition to the previously mentioned potential of visually classifying and staging strictures, direct visualization of the lesion can also allow for targeted destruction via radiofrequency ablation. SOC was used in one patient with recurrent complications from cholangiocarcinoma including bile duct stenosis and frequent stent occlusion. Cholangioscopy allowed for visually guided radiofrequency ablation in multiple occluded segments of the bile duct. The patient tolerated the procedure well and experienced no further strictures [32]. Another case was described with a patient found to have resectable intraductal papillary neoplasm of the bile duct. The patient opted for endoscopic treatment instead of surgery and cholangioscopy was used for targeted biopsy and radiofrequency ablation of the neoplasm. The patient was successfully treated and cholangioscopy was also used for follow-up surveillance of the lesion [33].

## 8. Evaluation of Hemobilia

Another area where cholangioscopy may be advantageous is in the diagnosis and treatment of hemobilia. Standard MRCP and ERCP can show the presence of blood in the bile duct, but they may not be able to reveal the cause of the bleeding and the underlying pathology. Cholangioscopy can be helpful to identify the exact source of bleeding and visual inspection of the tissue can aid in the diagnosis. One case describes a patient found to have hemobilia with no underlying pathology identified on MRCP. Cholangioscopy was then performed and direct visualization of the duct identified a lesion consistent with biliary angiodysplasia, a rare cause of hemobilia [34]. Another case described a patient with a suspected gallbladder malignancy and the presence of hemobilia. SOC was used and visualization of the bile duct confirmed that the hemobilia was due to the underlying malignancy and not a separate pathology [35].

## 9. Economic Factors

Cost is an important factor for the adoption of SOC as a viable option in patients with pancreatobiliary disease. Data on the economic costs of cholangioscopy are limited, but with the high technical success rates and low complication rates, SOC may also prove to be financially beneficial. The economic utility of SOC was assessed in a 2017 study evaluating which ERCP based technique was most cost effect for determining cholangiocarcinoma in patients with PSC. The study compared SOC guided targeted biopsy with common ERCP methods such as brush cytology, brushing with FISH, and ERCP guided biopsy. The cost-effectiveness and quality-adjusted life year was calculated for each method and cholangioscopy guided biopsy was found to be the most cost-effective option. ERCP with brush cytology was found to be the most commonly used method due to its low cost and ease of use, but SOC was the most cost-effective due to the high sensitivity in detecting cholangiocarcinoma [36].

A recent study evaluated if the usage of cholangioscopy in patients with difficult bile stones or indeterminate strictures would improve upon treatment and economic outcomes. The group looked at 111 total patients and assessed the total number of procedures required and the associated costs compared with using traditional ERCP methods. It was found that SOC resulted in a 27% reduction in the number of procedures and an 11% reduction in total costs for patients with difficult biliary stones. In patients with indeterminate strictures, SOC led to a 31% reduction in the number of procedures and an 11% reduction in costs [37]. The outcomes of this study indicate that the increased effectiveness when using SOC for diagnosis and treatment of difficult stones and indeterminate lesions does translate to improved economic outcomes.

## 10. Complications Associated with Cholangioscopy

In general, cholangioscopy is seen as safe, but studies have shown a higher rate of adverse events compared to ERCP based therapies. The most commonly reported complications are cholangitis, pancreatitis, and hemobilia [38]. SOC also comes with the potential for more serious complications such as perforation or air embolism [39]. While both SOC and ERCP have associated complications, it is important to assess and compare the safety profiles of each method. A recent retrospective study compared the adverse events seen after ERCP and SOC. It was found the SOC was associated with an adverse event rate of 7% compared to 2.9% in ERCP. SOC was also found to have a significantly higher rate of cholangitis compared to ERCP (1% vs. 0.2%). The authors theorized that this may be due to the use of intraductal irrigation during cholangioscopy leading to retrograde bacterial flow [40]. The group also evaluated adverse events seen after pancreatoscopy such as pancreatitis. The rate of adverse events was higher in patients after pancreatoscopy compared to ERCP techniques (7% vs. 3%), but rates of pancreatitis were similar between the two procedures (2.2% vs. 1.3%) [40].

A study in 2018 found similar results when looking at the complications associated with the use of cholangioscopy. The group found a complication rate of 13.2% after SOC with cholangitis being the most common complication (12.8%). However, if patients were given prophylactic antibiotics, the rate of cholangitis fell to 1%. The authors concluded that while there may be a higher risk of cholangitis after cholangioscopy, it is generally a safe procedure and administration of a single dose of antibiotics can be used to reduce the risk [41]. Further research is still needed in this area, and patients should be evaluated case-by-case based on their condition, indications for the procedure, and overall risk factors. 

## 11. Limitations of Single Operator Cholangioscopy (SOC) and Other Options

While the SpyGlass SOC system has helped drive the expanding use of cholangioscopy due to its ease of use, it does have limitations. The smaller diameter of the scope and smaller working channel limits image quality and accessory options. In addition to a lower image resolution, SpyGlass is also unable to use techniques such as chromoendoscopy and narrow band imaging [42]. Direct cholangioscopy offers superior image quality and also larger accessory tracts that support virtual chromoendoscopy [43]. However, it is limited by difficult maneuverability and technical difficulties entering smaller bile ducts [42]. While dual operator cholangioscopy is limited by its size and need for two operators, it does allow for the use of chromoendoscopy and narrow band imaging. This may offer the benefit of visualization of finer mucosal and vasculature detail that would otherwise not be appreciated using other techniques [3]. 

## 12. Conclusions

With the development of newer technology and easier to use instruments, single operator cholangioscopy is becoming increasingly utilized as an effective tool for patients with pancreatic and biliary pathology. Direct visualization of the bile duct has been shown to improve diagnostic and therapeutic outcomes, and early studies have also suggested that the therapy may be cost-effective. In addition, the technology is continuing to improve with the recent development of the third-generation SpyGlass DS II featuring even greater resolution [6]. Further large-scale studies can add to the current data by incorporating a greater number of patients as well as results from the use of newer digital SpyGlass systems. Overall, cholangioscopy is an exciting technology that may soon be a standard aspect of management in patients with pancreatic and biliary disease.

## Figures and Tables

**Table 1:** The progression of SpyGlass single operator cholangioscopy.

First Generation SpyGlass (2007)	SpyGlass DS (2015)
Single-operator system	Single-operator, single-use system
Fiberoptic picture with improved resolution	Digital sensor leading to 4× greater resolution
4-way tip deflection for increased maneuverability	60% wider field-of-view
Dedicated irrigation and accessory channel	Redesigned accessory channel for easier use

**Table 2:** The diagnostic and therapeutic benefits of cholangioscopy.

Diagnostic
Evaluation of strictures
Direct visualization of missed stones (from standard cholangiography)
Visually guided biopsy
Evaluation of hemobilia
**Therapeutic**
Visually guided electrohydraulic and laser lithotripsy
Visually assisted guidewire placement
Guided radiofrequency ablation of ductal tumors

**Table 3:** Established and investigational uses of cholangioscopy.

Established	Investigational
Difficult bile duct stones	Post liver transplant surveillance and stricture treatment
	Evaluation of hemobilia
Indeterminate biliary stricture	Radiofrequency ablation of tumors
	Guidewire placement in advanced strictures
	Staging of cholangiocarcinoma
	Primary Sclerosing Cholangitis (PSC) stricture evaluation
